# Oestrogens Downregulate Tissue Factor Pathway Inhibitor through Oestrogen Response Elements in the 5’-Flanking Region

**DOI:** 10.1371/journal.pone.0152114

**Published:** 2016-03-21

**Authors:** Huda Omar Ali, Benedicte Stavik, Christiane Filion Myklebust, Elisabeth Andersen, Anders E. A. Dahm, Nina Iversen, Per Morten Sandset, Grethe Skretting

**Affiliations:** 1 Department of Haematology, Oslo University Hospital, Oslo, Norway; 2 Research Institute of Internal Medicine, Oslo University Hospital, Oslo, Norway; 3 Institute of Clinical Medicine, University of Oslo, Oslo, Norway; 4 Department of Haematology, Akershus University Hospital, Nordbyhagen, Norway; 5 Department of Medical Genetics, Oslo University Hospital, Oslo, Norway; II Università di Napoli, ITALY

## Abstract

Oestrogens influence the pathology and development of hormone-sensitive breast cancers. Tissue factor pathway inhibitor (TFPI) has been shown to be associated with breast cancer pathogenesis. Recently, we found TFPI mRNA levels to be significantly reduced by oestrogens in a breast cancer cell line (MCF7), a process mediated through the oestrogen receptor alpha (ERα). The aim of the present study was to investigate the mechanism(s) by which oestrogens may regulate TFPI at the transcriptional level. The TFPI 5’-flanking region contains three oestrogen response element (ERE) half-sites at positions -845, -769 and -50. Constructs containing the wild type or mutated ERE half-sites of the TFPI 5’-flanking region were generated in a luciferase reporter gene vector and transiently co-transfected with an ERα expression vector into HEK293 cells and subsequently treated with oestrogens. We found that luciferase activity was significantly downregulated after oestrogen stimulation in cells transfected with the wild type construct, an effect that was abolished by mutating either ERE half-sites. Electrophoretic mobility shift assay suggested direct and specific interaction of ERα with the ERE half-sites in the TFPI 5’-flanking region. Chromatin immunoprecipitation showed that ERα was recruited to the region -899 to -578 of the TFPI 5’-flanking region *in vivo*, where the ERE half-sites -845 and -769 are located. Our results indicate that ERα can interact with all three ERE half-sites in the TFPI 5’-flanking region and thus participate in the repression of oestrogen mediated TFPI transcription in breast cancer cells.

## Introduction

Tissue factor (TF) pathway inhibitor (TFPI) is the main physiological inhibitor of TF induced coagulation [1]. The mature TFPI protein is 42 kDa in size and consists of an acidic N-terminal region, three tandem Kunitz-type serine protease inhibitor domains, and a basic C-terminus [2]. TFPI is mainly synthesized by endothelial cells, however, its expression has been detected in many cancer cells, in particular breast cancer cell lines [3] and circumstantial evidence suggests that it possesses anti-cancer properties [4].

Reduced plasma TFPI levels have been reported in healthy postmenopausal women receiving hormone replacement therapy [5], suggesting a possible link between oestrogens and TFPI. Oestrogens are known to influence the pathology of many hormone sensitive cancers, and in breast tumours they mainly exert their effect through ligand-dependent nuclear receptors that possess transcriptional ability. Oestrogen receptors (ERs) exist in different isoforms, namely ERα and ERβ, which are encoded by two separate genes located on different chromosomes [6,7]. Upon entering the target cells, oestrogen ligands bind to ERα, which enables dimerization of the oestrogen-bound ERα complexes and subsequent translocation into the nucleus. The ligand-bound ERα complex binds to specific DNA sequences known as oestrogen response elements (EREs) [8]. Approximately 50% of all ERα-bound loci have no discernible EREs, and most ERα-bound EREs are not perfect consensus EREs [9]. ERα dimers can also bind to ERE half-sites [10,11], and binding to an ERE can be enhanced by other transcription factors, such as specificity protein-1 (Sp1) [12,13], activator protein 1 (AP-1) [14], runt-related transcription factor 1 (RUNX1) [15], or nuclear factor-ĸB (NF-ĸB) [16].

In a recent report, we demonstrated that oestrogens downregulated TFPI mRNA and protein levels in the ERα expressing breast cancer cell line MCF7 [17]. This effect was mediated through ERα and the genomic pathway and was independent of de novo protein synthesis. The *TFPI* gene is located on chromosome 2 and consists of nine exons spanning 70kb [18]. Potential binding sites for various transcription factors, such as GATA-2, Sp1, c-Myc and AP-1, have been identified in the 5’-flanking region of the *TFPI* gene [19–22]. No studies have, however, yet reported possible oestrogenic regulation of the TFPI 5’-flanking region. Thus, the molecular mechanism(s) behind the oestrogen-mediated reduction of TFPI still remains to be elucidated. In the present study, we aimed to identify functional oestrogen responsive regions within the 5’-flanking region of the *TFPI* gene. The results indicate involvement of all three ERE half-sites in the oestrogenic regulation of TFPI expression.

## Materials and Methods

### Reagents

Dulbecco’s modified Eagle Medium (DMEM) with high glucose and L-glutamine, phenol-red free DMEM with high glucose without L-glutamine, charcoal dextran treated foetal bovine serum (FBS) and L-glutamine were purchased from the Lonza Group Ltd (Basel, Switzerland). Opti-MEM^®^ I Reduced Serum Medium was purchased from Thermo Fisher Scientific (Rockford, IL, USA). FBS for cell growth and phosphate-buffered saline (PBS) were purchased from GE Healthcare Life Sciences (Pittsburgh, PA, USA). 17α-ethinyl estradiol (EE2) was purchased from Sigma Aldrich (#E4876, St. Louis, MO, USA). 5X passive lysis buffer was purchased from Promega (Madison, WI, USA). A pCMV6-Neo vector containing the ERα cDNA (ESR1, SC125287) was purchased from ORIGENE (Rockville, MD, USA). Lipofectamine LTX with plus reagent for transfection was obtained from Invitrogen (Carlsbad, CA, USA). Bovine serum albumin (BSA) was purchased from New England BioLabs (Ipswich, MA, USA). siRNAs against c-JUN (ID 3725 Trilencer-27 siRNA SR302499) and Sp1 (ID 6667 Trilencer-27 siRNA SR304537) were purchased from ORIGENE, as was the Universal Scrambled Negative Control siRNA SR30004. Taqman assays for Sp1 (assay Hs00916521_m1) and c-JUN (qHsaCEP0032009) were from Applied Biosystems (Foster City, CA, USA) and Bio-Rad Laboratories (Hercules, CA, USA), respectively. INTERFERin transfection agent was purchased from Polyplus (New York, NY, USA),

### Cell lines and cultures

The human mammary adenocarcinoma MCF7 (ATCC^®^ HTB-22) and the human embryonic kidney HEK293 (ATCC^®^ CRL1573^™^) cell lines were obtained from the American Type Culture Collection (ATCC, Manassas, VA, USA) and cell line passages of <25 were used for all experiments. The cells were maintained in a humidified atmosphere with 5% CO_2_ in Dulbecco’s modified Eagle’s medium (DMEM) (Lonza Group Ltd., Basel, Switzerland) supplemented with 10% heat inactivated foetal bovine serum (Lonza Group Ltd.), 100 U mL^-1^ penicillin and 100 μg mL^-1^ streptomycin (Lonza Group Ltd.).

### Plasmid constructs

The luciferase reporter construct containing the 5’-flanking region of the *TFPI* gene spanning +28 to -1228 was generated as previously described [23]. Briefly, the 5’-flanking region was PCR amplified from human genomic DNA and subsequently cloned into the promoterless pGL3 Basic vector containing the luciferase cDNA (Promega, USA). The pGL3-TFPI wild type (pGL3-TFPI_wt_) construct was used as template to create additional luciferase reporter constructs with mutated EREs (TGACC replaced by GGGGG) using the Quick-change II Site-Directed Mutagenesis Kit (Stratagene, La Jolla, CA, USA). The mutated sequences were confirmed by sequencing. All oligonucleotides used for mutagenesis are listed in Table 1.

**Table 1:** Oligonucleotide sequences (5’-3’).

Name	Application	Sequences
TFPIfw_chip-473_	ChIP	CTGAGTAGCCAAGTTAGCAAGT
TFPIrev_chip-324_	ChIP	TGTTTCACCCACTGTCAATGT
EREfw_chip297_	ChIP	ACAGAGAGGTCTAAGGAAAGCA
ERE2rev_chip636_	ChIP	GGCAGGACCCAGCATGTA
EREfw_chip1091_	ChIP	TCCTTCATCTGTTTCCTCCACT
ERErev_chip1211_	ChIP	ACTGGCAGTTGGGAATAGCC
TFPI-fw3_Neg_	ChIP	AAAGCCTGACACCTGCAA
TFPI-rev3_Neg_	ChIP	CCCCTAGAGTTAGGGTTTA
EREfw_SDM-845_	SDM	CTCCTACATTTTCGGGGGAAATGTCTTCAGC
ERErev_SDM-845_	SDM	GCTGAAGACATTTCCCCCGAAAATGTAGGAG
EREfw_SDM-769_	SDM	GATCTTAGTCTGATAGGGGGATAATTCCAATACAG
ERE rev_SDM-769_	SDM	CTGTATTGGAATTATCCCCCTATCAGACTAAGATC
ERE fw_SDM-50_	SDM	GTCAGAGTTGCAGGGGGGTAAACAGGAAG
ERE rev_SDM-50_	SDM	CTTCCTGTTTACCCCCCTGCAACTCTGAC
ERE-845fw_wt_	EMSA	CATTTTCTGACCAAATGTCT
ERE-845rev_wt_	EMSA	AGACATTTGGTCAGAAAATC
ERE-769fw_wt_	EMSA	TCTGATATGACCATAATTCC
ERE-769rev_wt_	EMSA	GGAATTATGGTCATATCAGA
ERE-50fw_wt_	EMSA	GTTGCAGTGACCTAAACAGG
ERE-50rev_wt_	EMSA	CCTGTTTAGGTCACTGCAAC
ERE-845fw_mut_	EMSA	CATTTTCTGACCAAATGTCT
ERE-845rev_mut_	EMSA	AGACATTTGGTCAGAAAATC
ERE-769fw_mut_	EMSA	TCTGATATGACCATAATTCC
ERE-769rev_mut_	EMSA	GGAATTATGGTCATATCAGA
ERE3-50fw_mut_	EMSA	GTTGCAGTGACCTAAACAGG
ERE-50rev_mut_	EMSA	CCTGTTTAGGTCACTGCAAC

SDM; site-directed mutagenesis

### Transient transfections and luciferase reporter assays

For each experiment, 0.5 x 10^4^ HEK293 cells were seeded in 24-well plates in 500 μL phenol red-free DMEM with 10% v/v FBS treated with charcoal dextran. The cells were grown for up to 48 hours prior to co-transfection of 0.25 μg of each luciferase reporter construct with 0.25 μg of the pCMV6-Neo vector (ORIGENE) containing the ERα cDNA using 1 μL Lipofectamine LTX Reagent and Plus reagent (Invitrogen) according to the manufacturer’s instructions. As an internal control, the cells were also co-transfected with 5 ng of pRL-SV40 vector (Promega) containing the Renilla luciferase cDNA (hRluc) under the control of the Simian vacuolating virus 40 (SV40) promoter. Four hours after transfection, the cells were shifted into fresh phenol red-free medium and 24 hours post transfection, the cells were washed twice with PBS and shifted into serum and phenol red-free medium for another 24 hours before stimulated with 10 nM EE2 or vehicle (DMEM). Cells were then washed once with PBS and lysed in 175 μL of 1X passive lysis buffer (Promega). The luciferase activity was measured using the Dual-Luciferase Reporter Assay System (Promega) in a GloMax^®^-96 Microplate Luminometer reader (Promega) and corrected for Renilla internal control. Cignal ERE pathway reporter assay kit (QIAGEN, Valencia, CA, USA) was used to confirm the functional expression of ERα after transfection. For fold change, the luciferase output was normalized against the promoterless pGL3-Basic vector, and the luciferase activity from unstimulated transfected cells was arbitrarily defined as 1. At least three independent experiments were carried out for each construct. Results are expressed as mean + standard deviation (SD).

### RNA interference experiments

MCF7 cells (7x10^4^ cells/well) were seeded in 24-well plates, incubated at 37°C and subjected to transfection 24 h later. Human c-JUN and Sp1 was transiently knocked down with 1 nM c-JUN or Sp1 siRNA, respectively. Cells transfected with a 1 nM scrambled siRNA were used as a negative control. Transfections were performed using INTERFERin according to the manufacturer's instructions. 24 h post transfection, the medium was changed to phenol red-free DMEM with 10% v/v FBS treated with charcoal dextran and L-glutamine (4 mM). After 24 h the cells were exposed to EE2 (±10 nM) for additional 16 before harvesting for RNA isolation.

### Quantitative RT-PCR

The MagMax-96 Total RNA isolation kit and the ABI PrismTM 6100 Nucleic Acid PrepStation (Applied Biosystems) were used to isolate total RNA from the cells. The RNA yield was determined using the NanoDrop ND-8000 UV–Vis spectrophotometer (NanoDrop Technologies, Wilmington, DE). Reverse transcription was performed using the High Capacity cDNA Reverse Transcription Kit (Applied Biosystems) according to the manufacturer’s protocol. 1–50 ng cDNA was amplified using the Taqman Universal PCR master mix (Applied Biosystems) according to the manufacturer’s recommendations. The qRT-PCR reactions were carried out in triplicate using MicroAmp Optical 384 well plates on the ABI PRISM 7900 HT Sequence Detection System (Applied Biosystems) and the program was run at 50°C for 2 min, 95°C for 10 min, 40 cycles of 95°C for 15 sec and 60°C for 1 min. PMM1 was used as endogenous control and Ct values of the target genes were normalised against the endogenous control gene, which was not affected by the oestrogen treatment. Relative mRNA expression was calculated using the comparative threshold method (2^-ΔΔCt).

### Nucleic extracts and electrophoretic mobility shift assay (EMSA)

9x10^6^ MCF7 cells were seeded in a 14 cm dish in phenol red-free DMEM and allowed to grow for 24 hours before they were washed twice with PBS and subsequently shifted into serum and phenol red-free DMEM 16 hours prior to stimulation with either vehicle (phenol red-free DMEM) or 10 nM EE2 for 4 hours. Nuclear extracts were prepared using the NE-PER Nuclear and Cytoplasmic Extraction Reagents (Pierce Biotechnology Inc, Rockford, IL, USA) as previously described [24]. Protein concentrations were determined by use of the BCA protein-assay kit (Pierce). The biotin-labelled and corresponding unlabelled oligonucleotides spanning the ERE half-sites in the TFPI 5’-flanking region, the mutated ERE half-sites or the full ERE consensus sequence, were synthesized by Eurogentec (Seraing, Belgium), and are listed in Table 1. Equimolar amounts of complementary oligonucleotides were annealed to make double-stranded oligonucleotides. EMSA was performed using the LightShift Chemiluminescent EMSA kit (Pierce). Briefly, 3.5–5 μg of nuclear extracts were incubated with 20 fmol biotin-labelled oligonucleotides at 22°C for 20 min in 1X binding buffer in the presence of 1 μg μL^-1^ poly(dI-dC), 50 μM KCl, 1 μg μL^-1^ protein inhibitor cocktail (Sigma), and 0.1 μg μL^-1^ BSA (New England BioLabs). For competition experiments, 200–300 fold molar excess of corresponding unlabelled double stranded oligonucleotides were incubated with nuclear extracts for 40 min on ice before addition of 20 fmol biotin-labelled oligonucleotides. For supershift, 1–2 μg of ERα antibody (Santa Cruz Biotechnology Inc., Santa Cruz, CA, USA) was incubated with nuclear extracts at 4°C overnight prior to the addition of biotin-labelled oligonucleotides. DNA-protein complexes were then separated on a non-denaturing 5% polyacrylamide gel and transferred onto a positively charged nylon membrane (Pierce). The membrane was UV cross-linked, and the probes were visualized according to the LightShift Chemiluminescent EMSA kit (Pierce) and LAS 4000 Mini Imaging System (Fujifilm, Life Science, New Haven, CT, USA).

### Chromatin immunoprecipitation (ChIP)

9x10^6^ MCF7 cells were seeded in 14 cm dishes in 20–25 mL phenol red-free DMEM with high glucose supplemented with 4M L-glutamine and 10% v/v charcoal dextran treated FBS. and allowed to grow for up to 24 hours before starvation in phenol red-free DMEM without FBS 16 hours prior to cell treatment. 5x10^6^ HEK293 cells were seeded in 14 cm dishes in DMEM with 10% FBS and after two days the cells were transiently transfected with 17 μg of the pCMV6-Neo vector (ORIGENE) containing the ERα cDNA. 10 nM EE2 were added to the cells for 4 hours. For HEK293 cells, transfection of empty vector followed by EE2 treatment was included as a negative control. Chromatin immunoprecipitation (ChIP) was performed as previously described [25] with modifications. Briefly, proteins from cell extracts were cross-linked to DNA by addition of formaldehyde to a final concentration of 1% for 10 minutes at room temperature. A Biorupter (Diagenode, Belgium) was used to shear the chromatin so that the majority of fragments were 200–500 base pairs in size. The soluble chromatin fraction was collected, and 10% of the supernatant was used for input normalization. Equivalent amounts of anti-ERα antibody (sc-8002X, Santa Cruz Biotechnology) were added and incubated according to the protocol. The eluted and purified DNA was quantified by conventional PCR as described above. PCR primer sequences are provided in Table 1. The PCR reactions were carried out using Taq PCR Master Mix Kit (QIAGEN) following the manufacturer’s instructions and the PCR was run as follows: with the primer set covering the region 845 to -769: 94°C for 3 min, then 40 or 35 (MCF/ or HEK293, respectively) cycles at 94°C for 45 sec, 55°C for 45 sec, 72°C for 1 min followed by 72°C for 10 min; with the primer set covering the region -103 to +17: 94°C for 3 min, then 35 cycles at 94°C for 45 sec, 58°C for 45 sec, 72°C for 1 min followed by 72°C for 10 min.

### Bioinformatics Analysis of Transcription Factor Binding Sites

The prediction of oestrogen response elements and transcription factor binding sites in the TFPI 5’-flanking region spanning +28 to -1228 was performed with the program PROMO using the TRANSFAC version 8.3 and the cut-off for the dissimilarity rate set to 15% [26, 27].

### Statistical Analysis

Data distribution was confirmed by KS or Shapiro-Wilk normality test. One-way ANOVA with Bonferroni corrected tests for multiple comparisons was performed to test for significant differences between stimulated and unstimulated cells. For data that were not normally distributed, a non-parametric test (Kruskal-Wallis) was used. All statistical testing were conducted using GraphPad Prism 5.0 (GraphPad, San Diego, CA, USA) and a probability value (*p*) of ≤0.05 was considered significant.

## Results

### Prediction of EREs within the TFPI 5’-flanking region

To locate potential EREs, the TFPI 5’-flanking region spanning -1228 to +28 was analysed with the PROMO program. Using a dissimilarity rate of 15% no complete EREs were detected, however, the region contained three ERE half-sites (TGACC) at position -845, -769 and -50 relative to the transcriptional initiation site.

### Functional effects of oestrogen on TFPI transcriptional activity

**Fig 1:**
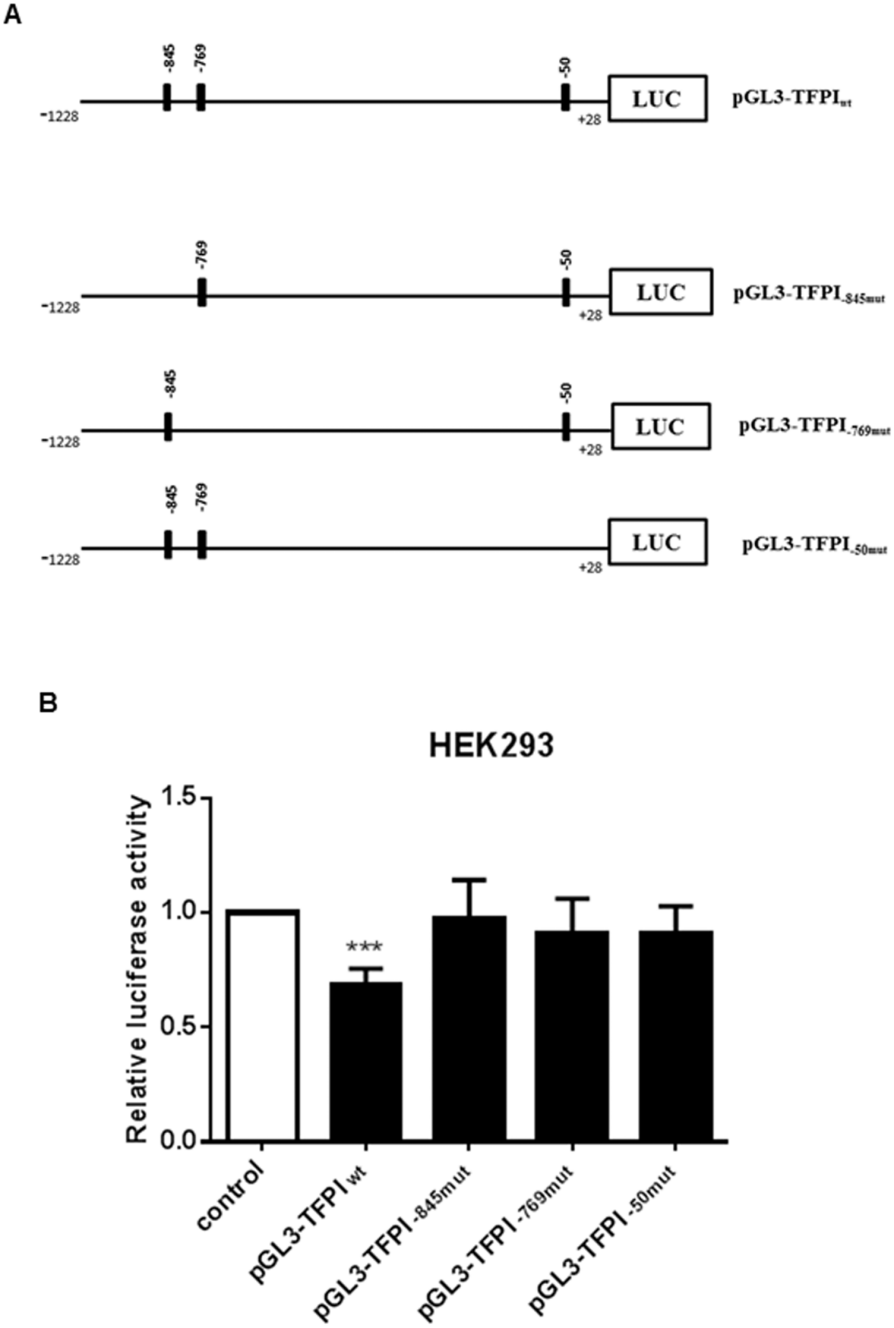
Transcriptional activity of the TFPI 5’-flanking region in response to oestrogens. (A) Schematic representation of ERE half-sites located in the TFPI 5’-flanking region. Construct containing the +28 to -1228 fragment of the TFPI 5’-flanking region (ERE half-sites located at -845, -769 and -50) was generated in the promoterless luciferase reporter vector pGL3-Basic (pGL3-TFPI_wt_) and was used to create constructs with mutated ERE half-sites using site-directed mutagenesis (pGL3-TFPI_-849mut_, pGL3-TFPI_-769mut_ or pGL3-TFPI_-50mut_). (B) HEK293 cells were co-transfected with ERα and constructs of the TFPI 5’-flanking region containing wild type or a mutated ERE half-site. The cells were treated with 10 nM EE2 for 24 hours and then analysed for luciferase activity. The luciferase activity was normalized against the renilla luciferase activity. The error bars represent standard deviation from at least three independent experiments (n≥9, *P<0.05 relative to untreated control cells).

To examine if oestrogens affected the TFPI expression through the ERE half-sites identified, a fragment spanning -1228 to +28 of the *TFPI* gene containing all three ERE half-sites was cloned into the promoterless luciferase reporter vector pGL3-Basic (Fig 1A, pGL3-TFPI_wt_). This construct was co-transfected with the pCMV6-Neo vector containing the cDNA for the ERα, also referred to as oestrogen receptor 1 (ESR1), into HEK293 cells and the luciferase activity was measured in response to oestrogen stimulation. A significant reduction in luciferase activity of approximately 30–40% was obtained with the pGL3-TFPI_wt_ construct in response to 10 nM EE2 as compared to vehicle stimulated cells (Fig 1B). To examine involvement of the ERE half-sites, each was mutated using site-directed mutagenesis. The resulting constructs (pGL3-TFPI_-845mut_, pGL3-TFPI_-769mut_ and pGL3-TFPI_-50mut_) are outlined in Fig 1A. Transfection analysis revealed that the effect of EE2 on the luciferase activity was abolished when each half-site was mutated (Fig 1B).

### Binding of ERα to ERE half-sites

**Fig 2:**
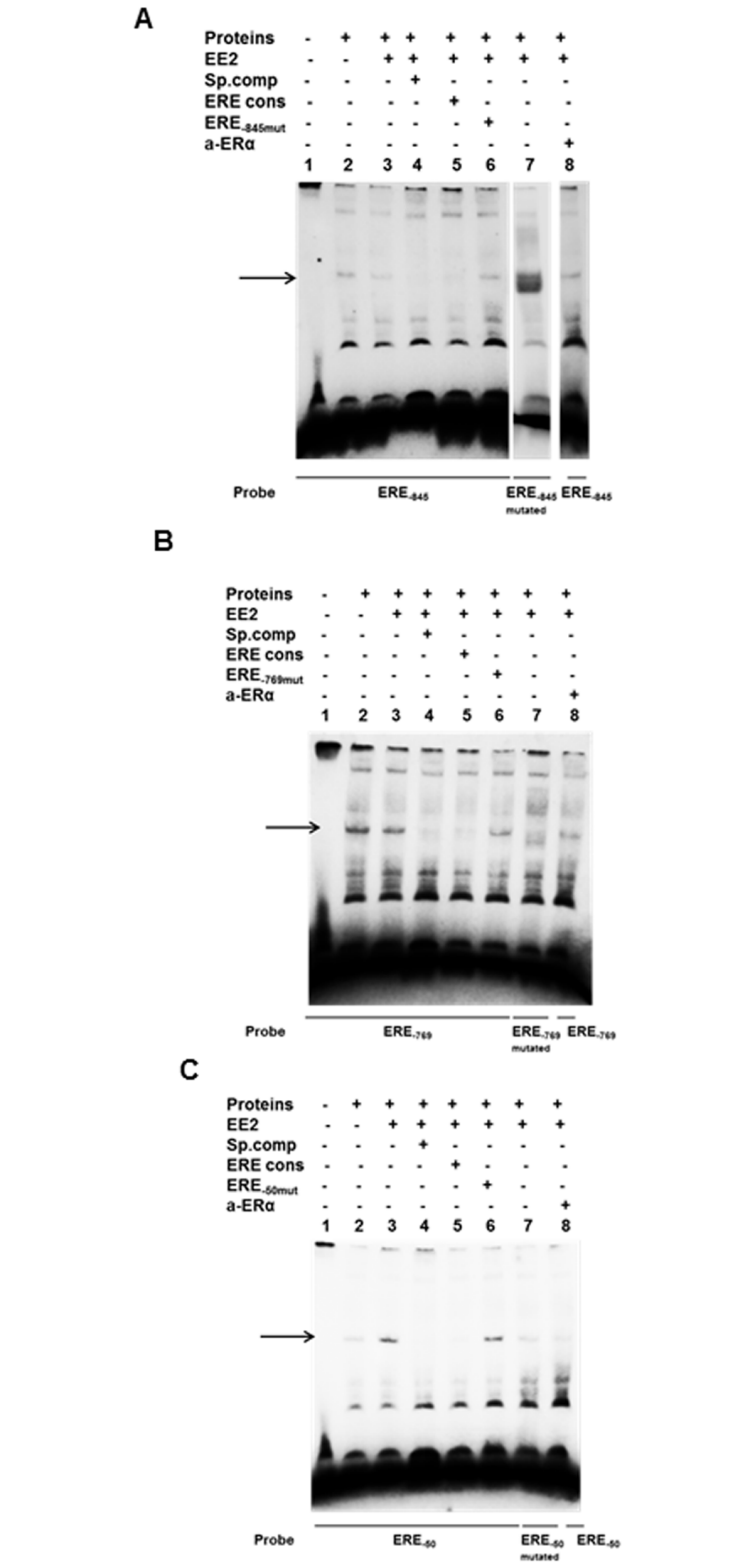
Binding of nuclear proteins to ERE half-sites of the TFPI gene by EMSA. MCF7 cells were treated ±10 nM EE2 for 4 h and nuclear proteins were isolated and incubated with biotin-labelled mutant or wild type oligonucleotides containing the ERE half-sites of interest as described in Table 1. (A) ERE half-site position -845, (B) ERE half-site position -769 and (C) ERE half-site position -50. The resulting complexes were resolved by non-denaturing polyacrylamide gel electrophoresis. For competition, a 200-fold excess of unlabelled specific (of corresponding sequence) or ERE consensus oligonucleotide were added during the pre-incubation period. The *arrows* indicate specific DNA-protein complex formation.

To examine potential interaction between ERα and the ERE half-sites identified, we performed electrophoretic mobility shift assay (EMSA). Double stranded biotin labelled probes containing the various ERE half-sites were incubated with nuclear extracts from MCF7 cells treated with ±10 nM of EE2 for four hours. To identify the migration of free probe no protein extract was added in lane 1 (Fig 2A–2C). Binding of nuclear proteins was detected with all three probes (Fig 2A–2C, lane 2). Increased binding of nuclear proteins was obtained with extracts from EE2 treated cells for the ERE half-site at position -50 compared to untreated cells (Fig 2C, lane 3 vs lane 2), whereas for the ERE half-sites at position -845 and -769, no such increase was detected (Fig 2A and 2B, lane 3 vs lane 2). For all ERE half-sites the binding was successfully competed with 200 fold molar excess of unlabelled corresponding oligonucleotides (Fig 2A–2C, lane 4). Moreover, successful competition with 200 fold molar excess of unlabelled oligonucleotide containing the full ERE consensus sequence was also observed (Fig 2A–2C, lane 5). To verify specific binding to the ERE half-sites, biotinylated oligonucleotide probes with mutated half-sites were tested with nuclear extract from EE2 treated cells. For the ERE half-sites at position -845 and -769 new complexes were generated in the same position (Fig 2A and 2B, lane 7), however, these complexes were not competed with unlabelled oligonucleotides containing the ERE half-site indicating them to be non-specific binding of proteins to the probes (data not shown). For the mutated half-site at position -50, a band was still detected (Fig 2C, lane 7), although much weaker compared to the one obtained with the non-mutated probe. To further confirm that the binding was specific, unlabelled oligonucleotides with mutated half-sites were used for competition with the biotin labelled wild type probe. The precise band was detectable (Fig 2A–2C, lane 6) confirming that specific binding required the intact TGACC motif. Moreover, the addition of anti-ERα resulted in reduced band intensity for the ERE half-sites -769 and -50 (Fig 2B and 2C, lane 8 compared to lane 3), further indicating binding of ERα to the TFPI 5’-flanking region.

**Fig 3:**
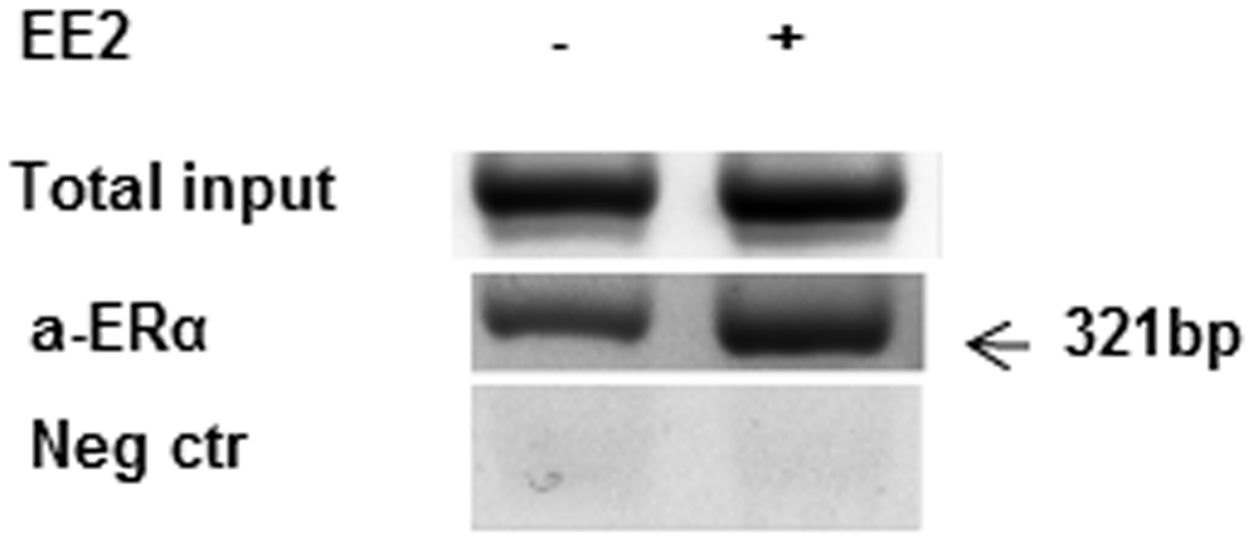
Recruitment of ERα to the TFPI 5’-flanking region *in vivo*. ChIP assays using anti-ERα antibodies were performed on chromatin isolated from MCF7 cells cultured in phenol red-free medium in the absence or presence of 10 nM EE2 for 4 hours. The equivalent fraction of the sonicated chromatin was set aside as 'input' DNA (non-immunoprecipitated) before the antibody affinity manipulations. The immunoprecipitated DNA and input was analyzed by conventional PCR with primers covering the ERE half-sites. For negative control, primers spanning a region without ERE half-sites were used. One representative result from two independent experiments is shown.

To verify that ERα dimers were recruited to the ERE half-sites *in vivo*, we next performed chromatin immunoprecipitation (ChIP) with MCF7 cells using an anti-ERα antibody. PCR amplification of the region -899 to -578 containing the distal ERE half-sites -845 and -769 yielded a PCR product of expected size (Fig 3). In contrast, no band was obtained when amplifying the region -103 to +17 containing the proximal ERE half-site at -50 (results not shown). As a negative control primers spanning a region without ERE half-sites were used for PCR. In addition, ChIP was performed with HEK293 cells transiently transfected to express ERα and subsequently treated with ±10 nM for 4 hours. Cells transfected with an empty vector and treated with 10 nM EE2 were used as a negative control. Similar results as for the MCF7 cells were obtained for the two distal ERE half-sites. As opposed to the MCF7 cells, we also observed a PCR product when the region containing the proximal ERE half-site, was amplified (S1 Fig).

### Involvement of Sp1 or AP-1 on the effects of oestrogen on TFPI expression

**Fig 4:**
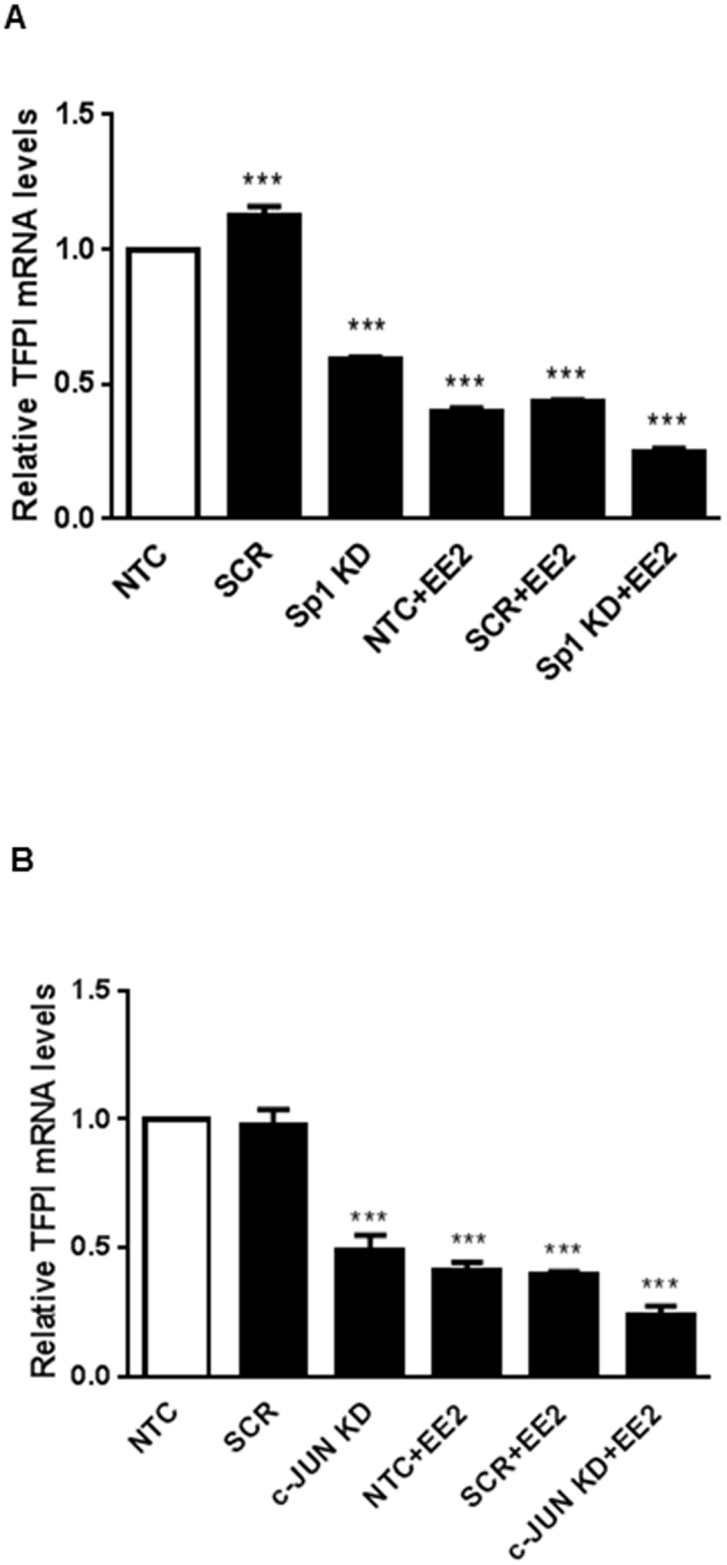
Knock down of Sp1 or AP-1. MCF7 cells were transfected with siRNA for Sp1 (A) or c-JUN (B). 24 hours after transfection, the cells were starved in phenol red-free medium for another 24 hours and thereafter treated with ± 10 nM EE2 for 16 hours before harvesting. mRNA levels were determined using qRT-PCR. Relative mRNA expression levels were calculated with the 2^-ΔΔCt^ method. The bars represent the mean relative mRNA expression levels after adjusting for the PMM1 endogenous control gene levels. The error bars represent standard deviation from three (A) or two (B) independent experiments with three biological parallels (*** ≤0.0001 relative to the non-transfected (NTC) cells).

We next examined whether Sp1 or AP-1 could be involved in the ERα mediated effect of oestrogen on TFPI expression. Sp1 or c-Jun that is one of the protein partners constituting AP-1, was transiently knocked down in MCF7 cells followed by stimulation with oestrogen. On average, a 30–40% knockdown of either transcription factor was achieved (S2 Fig). Knock down of both factors reduced the TFPI mRNA levels in itself. However, it did not abolish the effect of oestrogen on the TFPI mRNA levels (Fig 4A and 4B).

## Discussion

We have recently reported that oestrogens directly downregulated TFPI at the mRNA level, a process mediated by ERα and the genomic pathway [17]. In the present study, we have investigated possible molecular mechanisms behind this regulation by examining the TFPI 5’-flanking region using luciferase reporter gene assay, EMSA and ChIP analysis. We provide functional evidence that ERα was able to bind to ERE half-sites located in the 5’-flanking region of the *TFPI* gene and mediate the promoter activity of the *TFPI* gene. To our knowledge this is the first report describing a mechanism behind the transcriptional regulation of the *TFPI* gene by oestrogens in breast cancer cells.

ERα is known to signal through a genomic and a non-genomic pathway. During the classical genomic pathway, oestrogen ligands recognize and bind to ERα monomers, which leads to receptor dimerization. The complex is recruited to the promoter region of target genes and influences transcription through binding to EREs, whereas in the non-classical genomic pathway, ERα interacts with other transcription factors via their response elements [28]. Bioinformatics analysis of the TFPI 5’-flanking region did not reveal any complete ERE consensus sites, however, three ERE half-sites located at position -845, -769 and -50 relative to the transcriptional start site of the *TFPI* gene were identified.

In the present study, we aimed to examine the possible transcriptional regulation of the *TFPI* gene by oestrogens. Due to technical difficulties in transfecting MCF7 cells with large vectors such as the luciferase constructs, HEK293 cells with endogenous expression of TFPI were chosen as the experimental model for the luciferase reporter gene assay by co-transfecting with a vector containing the cDNA for ERα. The results from the luciferase reporter analysis indicated that oestrogens may regulate TFPI expression at the transcriptional level since reduced TFPI promoter activity in response to oestrogen treatment, was observed. Interestingly, mutation of each single ERE half-site was sufficient to eliminate the oestrogen mediated repression of TFPI promoter activity, suggesting that all three ERE half-sites must be present for complete regulation of *TFPI* gene transcription.

The human *TFPI* gene has been reported to contain several regulatory elements, which can regulate TFPI expression in various cell types. Negative regulatory elements have previously been identified in the TFPI 5’-flanking regions—75 to -390, -390 to -548 and -796 to -1158 in ECV30, HepG2 and THP1 cells, respectively, [19] and in the regions –345 to -446 and -446 to -1999 in HMEC-1 cells [19], in which both -845 and -769 ERE half-sites are present. To date, there has been no study indicating that oestrogens can regulate the transcriptional activity of the *TFPI* gene through regulatory elements in the TFPI 5’upstream region. ERα can bind ERE half-sites although often less strongly than the complete ERE consensus [29], and regulation of gene transcription by binding to a single or multiple ERE half-sites has previously been reported [11, 30, 31]. In our study, EMSA analysis showed specific binding to all three ERE half-sites following EE2 treatment, but it also revealed a specific complex formation with nucleic proteins from untreated cells. High affinity binding of oestrogen receptors to consensus EREs even in the absence of a ligand has previously been reported [30–32], which may explain the observed presence of ERα in the nuclei prior to EE2 stimuli. An increased specific complex formation following treatment with EE2 in MCF7 cells was seen with the proximal ERE half-site at position -50, but not with the two distal ERE half-sites.

Although ERα has traditionally been envisaged as a transcriptional activator, there are some studies indicating that transcriptional activators or repressors may adopt a multifaceted role [33]. In a recent report, ERα was indeed able to inhibit transcription of a gene through a single ERE half-site [34]. Our results indicate that ERα binds to all three ERE half-sites, all of which must be intact to repress *TFPI* gene transcription. These data alone cannot exclude the possibility that other transcription factors might interact with the ERE half-sites of the TFPI 5’-flanking region to repress transcription. ERα is known to interact with other transcription factors such as AP-1 and Sp1 [35–37], however, transiently knock down of either Sp1 or AP-1 (c-Jun) did not result in abolishment of the ERα mediated downregulation of TFPI mRNA levels. Furthermore, EMSA analysis of the TFPI 5’-flanking region spanning AP-1 or Sp1 binding sites did not reveal any specific complex formation following treatment with oestrogen for either transcription factor. Moreover, mutating the AP-1 binding site in the TFPI 5’-flanking region did not affect the luciferase activity after oestrogen treatment (unpublished observations). All together, these results strongly support that ERα binds directly to the ERE half-sites.

To confirm a direct interaction between the receptor and the TFPI 5’-flanking region, we assessed the *in vivo* recruitment of ERα to the ERE half-sites using ChIP with MCF7 cells. The results revealed enrichment of ERα in the TFPI 5’-flanking region between -899 and -578, which spans both distal ERE half-sites (-845 and -769), following EE2 treatment. Similar results were obtained in EE2 treated HEK293 cells transfected with the ERα. In contrast to the HEK293 cells, no visible ERα recruitment to the proximal ERE half-site (-50) was detected in the MCF7 cells. The divergent results between the two cell lines regarding the proximal ERE half-site might be due to technical issues. However, it might also reflect cell specific differences. Although ERα dimers are known to bind directly to ERE half-sites, recent reports have also indicated that other architectural proteins, such as high affinity mobility group protein B (HMGB1), may indeed enhance ERα mediated transcription by increasing the binding affinity between ERα with several ERE half-sites and consequently lead to increased transcription of target genes [10]. These multifunctional proteins activate transcription by binding to the minor grooves of DNA leading to an increased dynamic flexibility by facilitating bending of the DNA [38]. ERα dimers can also bend DNA by providing an allosteric effect on DNA to facilitate binding over larger distances. Our results may therefore suggest that ERα recruits directly to the distal ERE half-sites *in vivo*, but since both luciferase activity and EMSA results also indicated involvement of the proximal ERE half-site (-50), an indirect binding to this site may possibly be achieved through a DNA loop *in vivo* to facilitate ERα mediated suppression of TFPI transcription.

### Conclusions

Our results indicate that ERα binds to ERE half-sites in the TFPI 5’-flanking region at position -845, -769 and -50 relative to the transcriptional start site and suggest that all three ERE half-sites must be present together for oestrogen mediated repression of TFPI promoter activity and gene transcription to occur.

## Supporting Information

S1 Fig Recruitment of ERα to the TFPI 5’-flanking region in HEK293 cells. (PDF)

S2 Fig Knock down of Sp1 or AP-1. (PDF)
