# Supplementary material for: Oestrogens Downregulate Tissue Factor Pathway Inhibitor through Oestrogen Response Elements in the 5’-Flanking Region
Source: PLoS One. 2016 Mar 21;11(3):e0152114. doi: 10.1371/journal.pone.0152114 (PMC4801176; doi:10.1371/journal.pone.0152114)
Supplement: S1 Fig — (PDF) [file pone.0152114.s001.pdf]

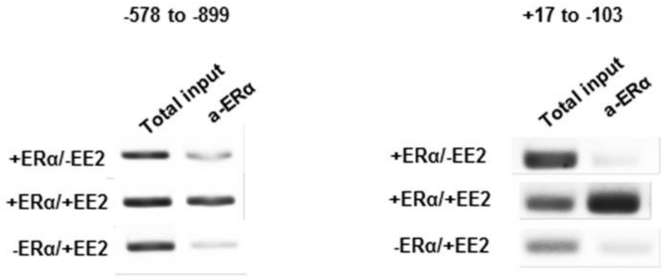

S1 Fig. Recruitment of ERα to the TFPI 5’-flanking region in HEK293 cells. ChIP assays using anti-ERα antibody were performed on chromatin isolated from HEK293 cells transfected with ± ERα cDNA and cultured in phenol red-free medium in the absence or presence of 10 nM EE2 for 4 hours. The equivalent fraction of the sonicated chromatin was set aside as 'input' DNA (non-immunoprecipitated) before the antibody affinity manipulations. The immunoprecipitated DNA and input were analyzed by conventional PCR with primers covering the ERE half-sites. One representative result from two independent experiments is shown.
