# Supplementary material for: Oestrogens Downregulate Tissue Factor Pathway Inhibitor through Oestrogen Response Elements in the 5’-Flanking Region
Source: PLoS One. 2016 Mar 21;11(3):e0152114. doi: 10.1371/journal.pone.0152114 (PMC4801176; doi:10.1371/journal.pone.0152114)
Supplement: S2 Fig — (PDF) [file pone.0152114.s002.pdf]

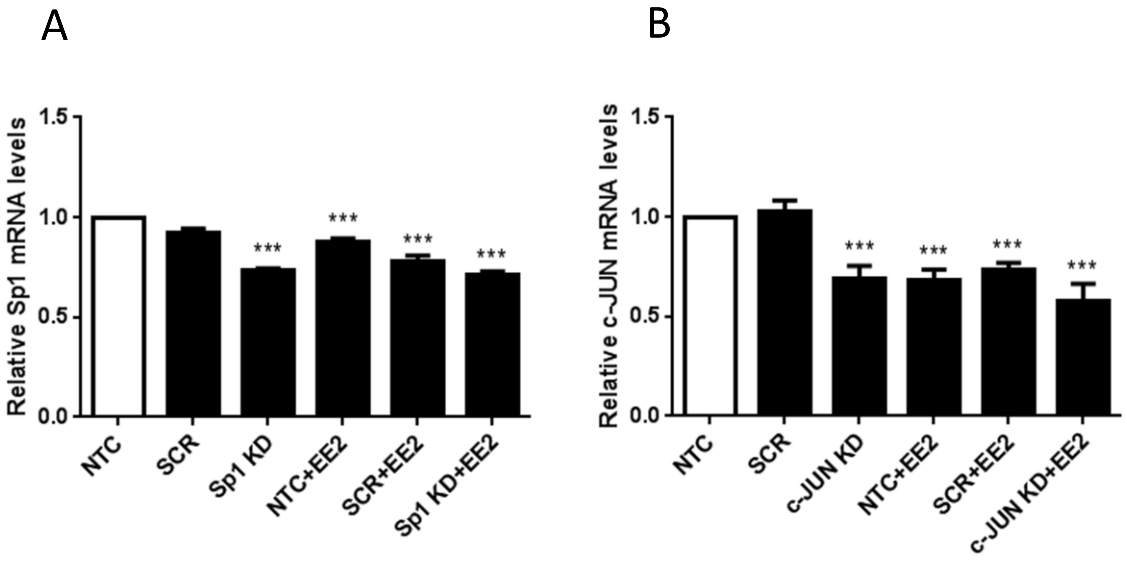

S2 Fig. Knock down of Sp1 or AP-1. MCF7 cells were transfected with 1 nM siRNA for Sp1 (A) or c-JUN (B). 24 hours after transfection, the cells were starved in phenol red-free medium for another 24 hours and thereafter treated with ± 10 nM EE2 for 16 hours before harvesting. mRNA levels were determined using qRT-PCR. Relative mRNA expression levels were calculated with the $2^{-\Delta\Delta Ct}$ method. The bars represent the mean relative mRNA expression levels after adjusting for the PMM1 endogenous control gene levels. The error bars represent standard deviation from three (A) or two (B) independent experiments with three biological parallels (***p≤0.0001 relative to the non-transfected (NTC) cells).
